# Mixed reality navigation training system for liver surgery based on a high‐definition human cross‐sectional anatomy data set

**DOI:** 10.1002/cam4.5583

**Published:** 2023-01-06

**Authors:** Muhammad Shahbaz, Huachun Miao, Zeeshan Farhaj, Xin Gong, Sun Weikai, Wenqing Dong, Niu Jun, Liu Shuwei, Dexin Yu

**Affiliations:** ^1^ Department of Radiology, Qilu Hospital of Shandong University Jinan Shandong China; ^2^ Research Center for Sectional and Imaging Anatomy Digital Human Institute, School of Basic Medical Science, Shandong University Jinan Shandong China; ^3^ Department of General Surgery Qilu Hospital of Shandong University Jinan Shandong China; ^4^ Department of Anatomy, Wannan Medical College Wuhu Anhui China; ^5^ Department of Cardiovascular Surgery, Shandong Qianfoshan Hospital, Cheeloo College of Medicine Shandong University Jinan Shandong China

**Keywords:** intrahepatic duct systems, liver, mixed reality model, three‐dimensional visualization, training and teaching system

## Abstract

**Objectives:**

This study aims to use the three‐dimensional (3D) mixed‐reality model of liver, entailing complex intrahepatic systems and to deeply study the anatomical structures and to promote the training, diagnosis and treatment of liver diseases.

**Methods:**

Vascular perfusion human specimens were used for thin‐layer frozen milling to obtain liver cross‐sections. The 104‐megapixel‐high‐definition cross sectional data set was established and registered to achieve structure identification and manual segmentation. The digital model was reconstructed and data was used to print a 3D hepatic model. The model was combined with HoloLens mixed reality technology to reflect the complex relationships of intrahepatic systems. We simulated 3D patient specific anatomy for identification and preoperative planning, conducted a questionnaire survey, and evaluated the results.

**Results:**

The 3D digital model and 1:1 transparent and colored model of liver established truly reflected intrahepatic vessels and their complex relationships. The reconstructed model imported into HoloLens could be accurately matched with the 3D model. Only 7.7% participants could identify accessory hepatic veins. The depth and spatial‐relationship of intrahepatic structures were better understandable for 92%. The 100%, 84.6%, 69% and 84% believed the 3D models were useful in planning, safer surgical paths, reducing intraoperative complications and training of young surgeons respectively.

**Conclusions:**

A detailed 3D model can be reconstructed using the higher quality cross‐sectional anatomical data set. When combined with 3D printing and HoloLens technology, a novel hybrid‐reality navigation‐training system for liver surgery is created. Mixed Reality training is a worthy alternative to provide 3D information to clinicians and its possible application in surgery. This conclusion was obtained based on a questionnaire and evaluation. Surgeons with extensive experience in surgical operations perceived in the questionnaire that this technology might be useful in liver surgery, would help in precise preoperative planning, accurate intraoperative identification, and reduction of hepatic injury.

## Graphical abstract

To Use the 3D mixed‐reality model of liver, entailing complex intrahepatic systems and to deeply study the anatomical structures and to promote the training, diagnosis and treatment of liver diseases. The higher quality 2D human liver cross‐sectional anatomical data set is used to construct a detailed three‐dimensional model. The resolution of the 2D data set reach 13,000 × 8000 pixels and the accuracy reach 416 million pixels, a great improvement compared with other studies. 3D printing & HoloLens technology, MRNSALTS hybrid‐reality navigation‐training system for liver surgery help in precise preoperative planning, accurate intraoperative identification, and reduction of hepatic injury and improves the traditional training methods of liver surgery.

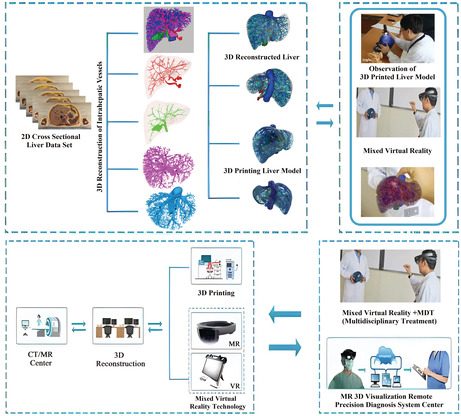

## 1 INTRODUCTION

Clinically, liver‐related diseases are relatively common, and the primary liver cancer is the second most common cause of death worldwide.^1^ Comprehensive treatment based on surgical resection of the tumor is the most effective for liver cancer.^2^ However, due to the complexity, variation of intrahepatic blood vessels, bile ducts branches, and the close relationship between tumors and those vessels, and ducts, hepatic surgery is challenging.

Imaging is the routine diagnosis and treatment assessment method of liver cancer. At present, the computer three‐dimensional (3D) reconstruction technology is gradually becoming mature, with a range from definite diagnosis, preoperative surgical planning, intraoperative navigation training, and even “last minute simulation”,^3^, ^4^ which can help doctors to intuitively grasp the fine details.

Since the Visible Human Project (VHP) appeared in 1995, several studies on 3D reconstruction of virtual liver based on tomographic anatomical datasets have been conducted.^5^, ^6^, ^7^, ^8^, ^9^ Advances in sectional anatomy are reflected in the maturity of specimen preprocessing technology, accuracy of milling, camera resolution, and the improvement of software and hardware related to image registration, segmentation and reconstruction.^10^ Thus, the 3D reconstructed virtual liver system based on cross‐sectional anatomical datasets can recreate, complete and meticulous model, which can provide a realistic anatomical and morphological theoretical basis for precise surgery. Moreover, with the development of 3D printing, the advantages of no mold and short production cycles, it is particularly suitable for the rapid delivery of complex structured and customized medical products. Due to its high degree of simulation and homogeneity, the model is well suited to help surgeons understand the configuration of complex organs and structures. Solid models printed by 3D technology can be used as tools for preoperative communication, visualization of complex structures, and surgical rehearsal and planning.^11^ This may reduce the surgical risks and improve surgical treatment outcomes.^12^

The 3D printing technology can help simulate surgery and improve the accuracy of surgery, but it cannot determine the course of intrahepatic vasculature, bile ducts and the location of the disease in real time intraoperatively. Hybrid or Mixed reality technology is an emerging holographic image technology that can realize the interaction between virtual world and reality. It combines the virtual and physical world to enhance the user's sense of reality. Due to this blending, MR is also called hybrid reality or extended reality. In this form of virtual reality, users can interact with both the physical and virtual worlds simultaneously.^13^, ^14^, ^15^ By combining 3D printing and mixed reality technology, it helps hepatobiliary surgeons to implement virtual data on the operating table in real time and accurately. The aim of the study is to establish a novel mixed reality technology to investigate and analyze its feasibility and application value in hepatic surgery. [Video Abstract].

The purpose of our system training is to make surgeons more proficient in intraoperative environment using mixed reality technology to assist them in making judgments and interpret and act upon any apparent error during surgery, a judgment that requires a combination of factors including prediction of the tissue deformation and use of advanced visualization algorithms to assist the surgeons to identify the overlay error. MR technology can help physicians to perform precise positioning during surgery. However, MR technology involves complex operations and physicians' proficiency in operating MR glasses limits the spread of this technology. 3D printing and MR technology can both help physicians perform accurate positioning during surgery. Our system gives doctors an opportunity to validate imaging data directly on the patient's body in a simulated surgery, which is of great interest to liver surgeons, and is believed to help in precise preoperative planning, accurate intraoperative identification, reduction of liver damage, and improvement of traditional liver surgery training methods (face validity, content validity, or construct validity).^4^, ^16^

## 2 MATERIALS AND METHODS

### 2.1 Acquisition of high‐definition two‐dimensional cross‐sectional images

The specimens selected in this study were obtained from fresh‐frozen cadaver from an adult female, (50 years old, height 160 cm, weight 62 kg, general condition was good, without underlying diseases). The magnetic resonance imaging showed the integrity of liver and peripheral structures, without corruption and liquefaction. After pretreatment, the blood vessels were exposed, vascular intubation and large blood vessel irrigation were performed. The arterial or venous fillers were made with gelatin plus red dye or blue dye respectively. In the supine position, a special embedding box was used and frozen with 5% gelatin solution. Stored in −25°C freezer for 2 weeks until frozen.

Full refrigeration fan set to −25°C, and 4 LED light sources, kept always bright, we used computer‐numerical‐control‐milling machine (Hanchuan XK2408B, China) to cut layer by layer from foot to head. Using the closed‐loop computer‐numerical‐control operating system, the milling thickness was set to 100 μm. In the process of milling, the RENCAY scanning system (Rencay 16 k3 Scanback) was used to scan and take pictures layer by layer, and the resolution was 13,000 × 8000 pixels. Through the large format mobile scanning system, the milling section of the specimen was transformed into two‐dimensional (2D) dataset. This study was approved by the Ethics Committee of Basic Medical Sciences, Shandong University (IRB No. ECSBMSSDU2018‐1‐050 and Ethics Committee of Scientific Research of Shandong University Qilu Hospital IRB No. KYLL‐2022(ZM)‐749).

### 2.2 Image processing and 3D model reconstruction

**FIGURE 1:**
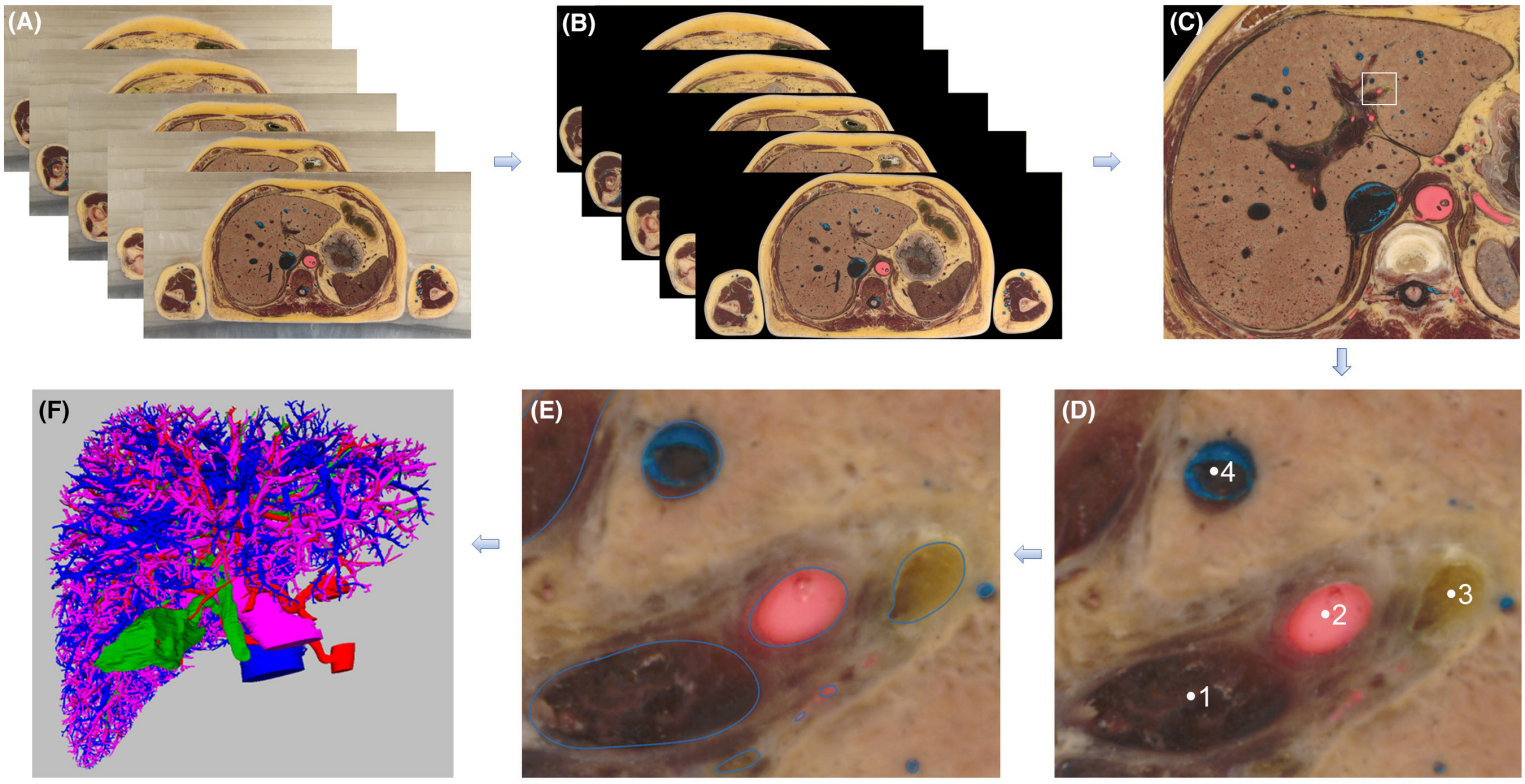
Image processing and reconstruction: (A) 2D Cross sectional Dataset of liver, (B) Image registration and deicing, (C) Liver in image No. 11277, (D) Partial enlarged view (1. Hepatic Portal Vein, 2. Proper Hepatic Artery, 3. Hepatic Duct, 4. Hepatic vein), (E) Path marking, (F) 3D reconstructed model.

The images in the 2D dataset were pre‐processed by the digital human specimen sectional sequence image processing system (Shandong Digihuman company). That included color calibration, deicing, brightness registration, spatial registration, etc. (Figure 1). Then the liver and duct systems on each 2D image were manually labeled. Each image was set up with five groups of paths. The liver shape group marked the main body outline of the liver on each level. The duct system was divided into (1) hepatic vein group (including inferior vena cava, and intrahepatic branches of hepatic vein), (2) hepatic portal vein group and branches, (3) proper hepatic artery and branchs, and (4) hepatic duct group (including gallbladder, cystic duct, common bile duct, common hepatic duct and branches). We manually segmented the shape and intrahepatic structures one by one using the Adobe Photoshop CC software 2018 (version 19) (Adobe Inc.).

After the data segmentation was completed, the digital human specimen sectional 3D reconstruction system (Shandong Digihuman company) was used to extract the manually segmented structural path according to the path components. The 3D reconstruction of its face was then drawn. The reconstructed file was in ply format. The model file was imported into MeshlabV2016.12 software for smoothing and simplifying the model surface. the interactive 3D model was then constructed. According to the results of 3D reconstruction, the structural composition, 3D morphology and adjacent relationship of the liver and its duct systems were observed and studied by sectional anatomy and 3D morphology.

### 2.3 3D printing of liver model

The normal model data were converted into STL format data that can be read by the 3D printer. The 3D printer (Sailner, model J401Pro, Zhuhai Sailner Company) was used to print the 1:1 simulation model. After setting the structural color of each part, we printed the model with PolyJet photopolymer (RGD720). The material used for 3D printing was photosensitive resin, which was developed and supplied by Medical Independent Research and Development Center of Shanghai Black Flame Medical Technology Co., Ltd.

### 2.4 Liver hybrid reality navigation training system

The 3D reconstructed liver data were imported into Microsoft Hololens (Microsoft, USA) to construct a liver hybrid display surgical simulation system (Anhui Ziwei King Star Digtal S&T Co., Ltd./VisionTeke Medical Imaging Systems Co., Ltd). Microsoft's head‐mounted holographic glasses Microsoft Hololens have spatial positioning and motion capture gesture operation functions, which can achieve automatic contour line tracking and attachment fusion of solid print models. Automatic tracking and fusion attachment is calculated through the contour line to achieve automatic identification of fusion adsorption, with the virtual model through gesture manipulation mobile rotation zoom in and out and other manipulation.^17^ A volunteer stood with a model liver placed on the right upper abdominal quadrant. The volunteer only needs to hold the 3D printed model to maintain a fixed position, the person wearing the Hololens helmet through gestures to manipulate the model display and positioning within the glasses, start automatic virtual reality fusion or manual manipulation of the mobile model and the model carried by the volunteer for alignment fusion, multi‐directional multi‐angle adjustment.^18^

Subjects wore HoloLens glasses and verified the position of intrahepatic structures on liver model under the guidance of HoloLens navigation glasses. The transparency of PolyJet photopolymer was high. The subject could verify overlapping important ducts in real time and maximize matching accuracy, so as to train for navigation through HoloLens glasses intraoperatively.^19^

### 2.5 Evaluation of its application in clinical teaching and training

Participants were recruited to participate in the “ Mixed Reality Navigation in Liver Surgical Anatomy: Learning and Training System”. After the training, the questionnaire was filled out by participant. The recruitment criteria were: (1) currently specializes in abdominal surgery, liver surgery and/or the treatment of liver diseases; (2) is currently a medical practitioner in this specialty; (3) is located in Jinan, Shandong Province.

An open questionnaire was designed for participants as the main body. The three main indicators of the questionnaire were: (1) Basic information of participants; (2) Perceptions related to clinical anatomy learning based on 3D printed liver models; (3) Perceptions related to mixed reality navigation training system in liver surgery. The participants filled in a questionnaire to investigate the training effect and the recognition of mixed reality technology for liver surgery. The recruited participants of “Mixed Reality Navigation in Liver Surgical Anatomy Learning and Training System,” were all clinically experienced liver surgeons, and the application of 3D printed models recruited a diverse group of liver surgeons. It does not vary from hybrid‐reality navigation training system. The training involved the know‐how of the basics of the HoloLens II, setting up and functions of the HoloLens II, and use HoloLens II navigation to verify the location of intrahepatic structures on a 3D‐printed liver model. It was conducted in hospital settings by filling out the questionnaire after the training.

## 3 RESULTS

### 3.1 2D images

We successfully obtained high‐resolution 2D image datasets of liver sections (100 μm). Taking the highest point and the lowest point of the liver as the boundary, the distance between them was 15.4 cm, a total of 1540 sections were obtained (Number 10480–12020). The magnified image was clear and undistorted. The structures were easy to identify. The color restoration was accurate. The collection process was continuous and there was no defect or deletion of the cross‐section. The four types of ducts in respective colors were clearly discernible (Figure 1).

### 3.2 3D reconstruction

After the manual segmentation of 1540 images, we used Digihuman 3D Reconstruction System to process five groups of paths to get five 3D model files, including liver, hepatic vein, hepatic portal vein, proper hepatic artery and hepatic duct groups. After the five 3D model files were modified with MeshLab software, each group of models were displayed separately or together in different colors, zoomed arbitrarily, rotated and observed at different angles. The visual image structure after 3D reconstruction was obvious and clear. The stroke and branches of the ducts truly reproduced the positional relationship and close relationship between the ducts and the liver (Figure S2, S3).

**FIGURE 2:**
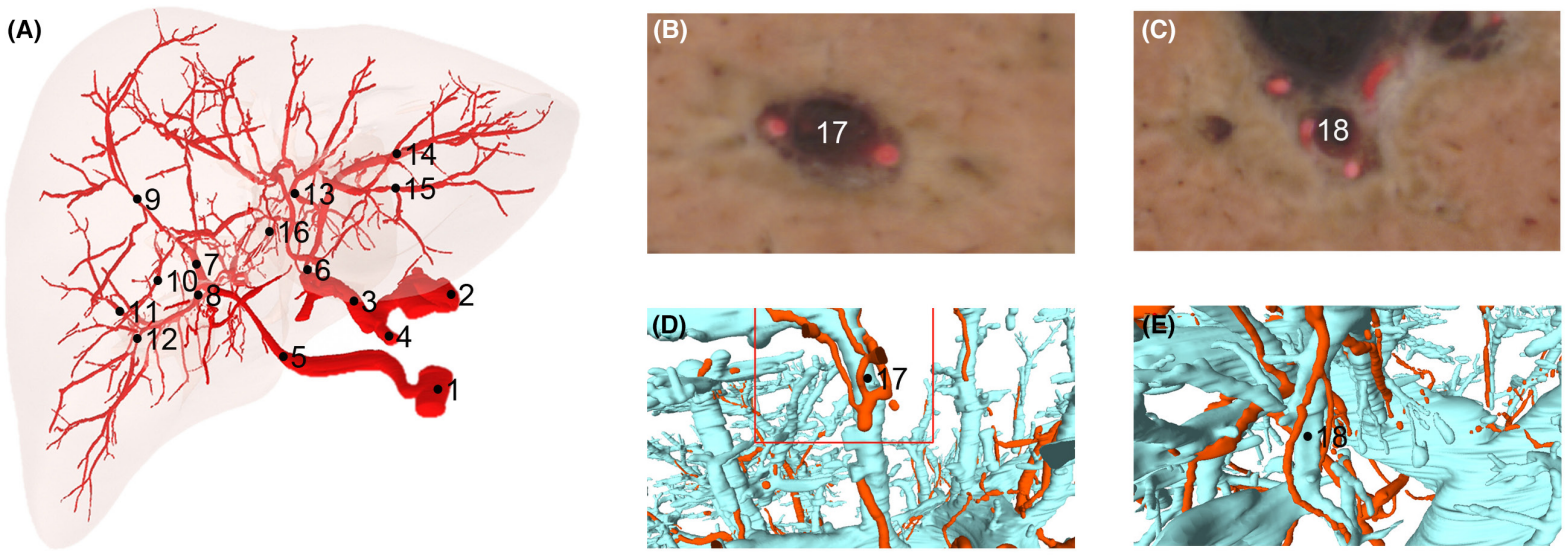
The reconstructed hepatic artery: (A) hepatic arteries 1. superior mesenteric artery, 2. celiac trunk, 3. proper hepatic artery, 4. gastroduodenal artery, 5. right hepatic artery, 6. left hepatic artery, 7. right anterior branch, 8. right posterior branch, 9. right anterosuperior branch, 10. right anteroinferior branch, 11. right posterosuperior branch, 12. right posteroinferior branch, 13. left medial branch, 14. left laterosuperior branch, 15. left lateroinferior branch, 16. Caudate branches). (B) The right anterior superior branch of the hepatic portal vein in transverse section. (C) Caudate branch of hepatic portal vein in transverse section. (D) The right anterior superior branch of the hepatic portal vein in reconstructed model. (E) Caudate branch of hepatic portal vein in transverse section.

**FIGURE 3:**
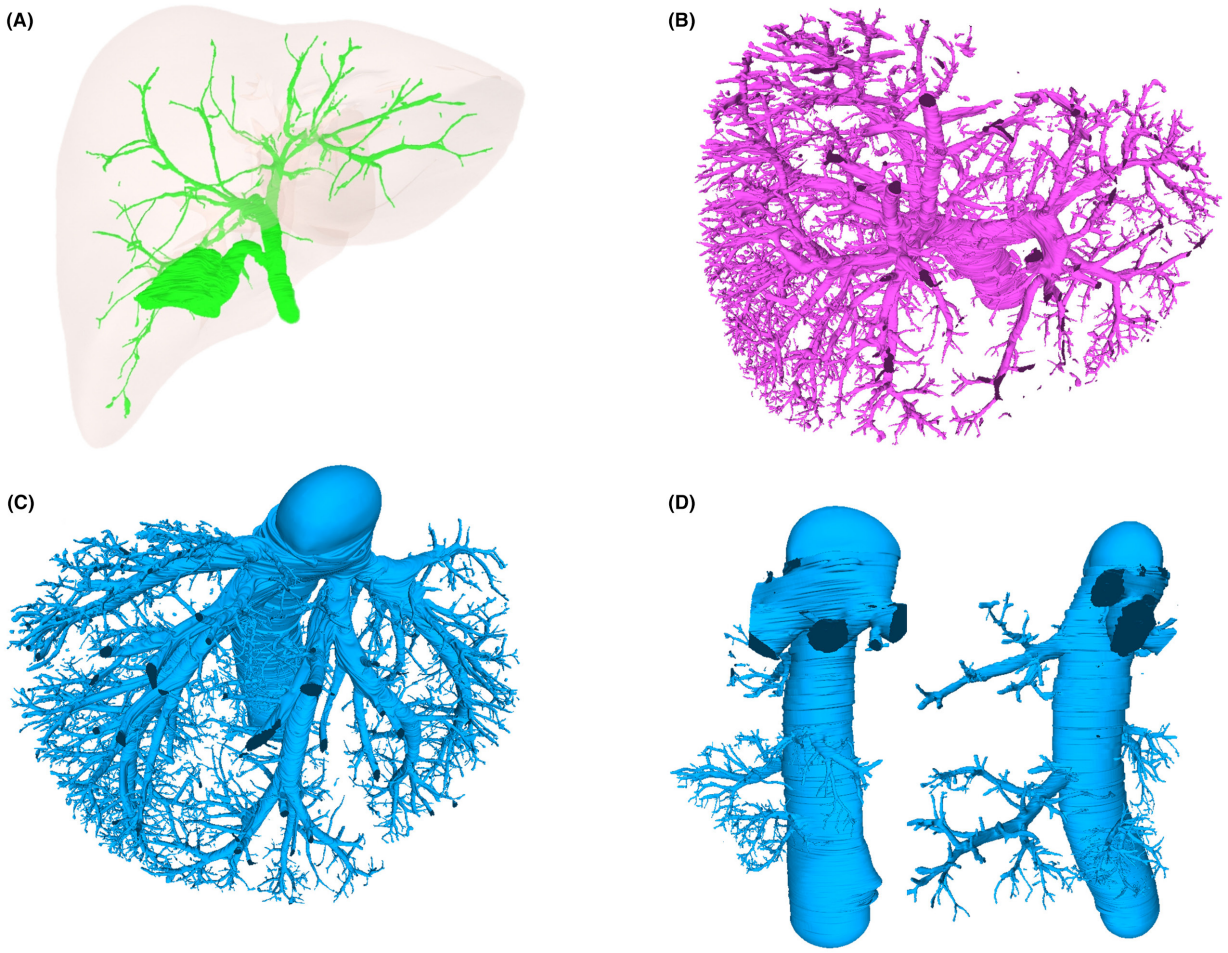
The reconstructed model of Hepatic duct: (A) Reconstruction of bile duct. (B) Reconstruction of hepatic portal vein. (C) Reconstruction of hepatic vein. (D) Reconstruction of accessary hepatic vein.

The left and right branches (lobe and segment artery), origin and distribution of the hepatic artery were displayed clearly (Figure 3A, Figure S2). Through the observation of the 3D structure of 3D reconstruction, it was found that the branches of the proper hepatic artery appeared in advance in 5 places. As shown in Figure 2B–E, around the branch of the right superior segment of the hepatic portal vein and the caudate lobe branch, there were 2–3 proper hepatic arteries around a hepatic portal vein. The shape of the hepatic duct accords with the common morphology. (Figure 3A, Figure S2). In addition, the hepatic portal vein was consistent with the normal type in the classification of hepatic portal vein of Couinaud (Figure 3B, Figure S3).

Moreover, the branches, confluence, distribution, formation of hepatic veins were also very distinct (Figure 3C). 6 accessory right hepatic veins were directly draining into the inferior vena cava (Figure 3D, Figure S4).

### 3.3 3D printed results

**FIGURE 4:**
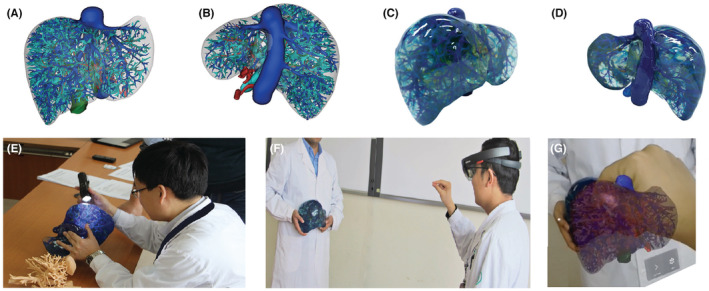
3D printed liver model: (A, B) data model before input of 3D printer. (C, D) Printed Liver model printed. (E) Clinical anatomy study of liver. (F) Wearing HoloLens II glasses for clinical teaching and training. (G) the field of vision obtained through HoloLens II glasses.

After entering the data into the Sailner J401 Pro 3D printer, in 12 h and 42 min, we printed out a full‐color transparent liver weighing 2743 g. The whole body was 15.4 cm × 18.9 cm height and width respectively. This accorded with the original size of the specimen. The internal duct system was clearly discernible (Figure 4). Data segmentation was performed by manual segmentation by two researchers, and it took 15,400 min/256.67 h (10 min per slice, for a total of 1540 slices). 3D printed material cost $0.50/g and weighed 2743 g. The total cost was US$1372. Model printing took 12 h and 42 min, and model post‐printing processing took 6 h.

### 3.4 Liver hybrid reality navigation training system

The 3D reconstructed liver data were imported into Microsoft HoloLens II (Microsoft, USA), and combined with the 3D printed model. The intraoperative navigation simulation was performed. We invited clinicians to wear HoloLens II glasses and use HoloLens II navigation to verify the location of intrahepatic structures on a 3D‐printed liver model. This improved the surgical navigation skills through HoloLens II glasses (Figure 4F,G).

### 3.5 Application in clinical teaching and training

Our mixed reality navigation training system is a combination of 3D printing and MR to simulate surgical scenarios for training and improve physician proficiency in MR technology. According to the recruitment criteria, 26 clinicians participated in the training of “Mixed Reality Navigation in Liver Surgical Anatomy: Learning and Training System”. After the training, we conducted a questionnaire evaluation. All participants were licensed liver surgeons (Table 1).

**TABLE 1:** Basic information of participants

Average age	33.9
Male ratio	84.6%
Liver surgeons	100%
Liver surgery localization and navigation	
CT/MRI	100%
3D reconstruction	69.2%
3D printing	15.4%
Mixed reality	0%

Abbreviations: CT, Computed tomography; MRI, Magnetic resonance Imaging; 3D, Three‐dimensional.

Among the questions related to clinical anatomy learning based on 3D printed liver model, we first set up a test question to ask how many accessory hepatic veins were in the model liver. The correct rate of this question was very low, and only 2 senior doctors answered correctly. There were 6 accessory hepatic veins in our model, of which two larger ones dominate the right posterior lobe, these two were obvious, and there were 4 in the right anterior and left anterior.

In the remaining questions, all agreed that it was very important to understand the hierarchical and spatial relationships of intrahepatic structures (including blood vessels and tumors) in the surgical plan for liver lesions. The complete outcome of the survey is stated in Table 2.

**TABLE 2:** Evaluation of problems related to clinical anatomy learning based on 3D printed liver model

Question	Count	Rate (%)
1. How many accessory hepatic veins do you see in the model?		
2	14	53.85
4	10	38.46
6	2	7.69
8	0	0
10	0	0
2. According to the distribution of intrahepatic ducts, can you segment the model liver based on these ducts and signature structure?		
Yes	26	100
No	0	0
3. Can 3D printed models give you a better understanding of the depth of intrahepatic structures?		
Yes	24	92.31
No	0	0
The effect is similar	2	7.69
4. Can 3D printed models give you a better understanding of the spatial relationships between the internal structures of the liver?		
Yes	24	92.31
No	2	7.69
The effect is similar	0	0
5. Is it important to understand the hierarchical and spatial relationships of intrahepatic structures, including blood vessels and tumors, in the surgical planning of liver lesions?		
1. Yes, information about hierarchical and spatial relationships is important	24	92.31
2. Yes, there are only structural levels	0	0
3. Yes, only the spatial relationships between structures	2	7.69
4. No, neither is important in the surgical plan.	0	0
6. Are independent 3D‐printed models useful in treatment planning or surgical procedures?		
Yes	26	100
No	0	0
7. Can 3D printed models help you determine safer surgical paths and improve your surgical plans?		
Yes	22	84.62
No	0	0
I'm not sure	4	15.38
8. Can 3D printed models help shorten surgical time by replacing the use of intraoperative visualization tools such as Doppler ultrasound and/or cholangiography?		
1. Yes	12	46.15
2. No	2	7.69
3. Not sure	8	30.77
4. Perhaps, depending on the complexity of the patient's pathology	4	15.38
9. Do you think it is possible to reduce intraoperative complications with 3D printed liver models?		
1. Yes, in all cases (complex and relatively simple cases)	6	23.08
2. Yes, only in complicated cases	18	69.23
3. Yes, only in simple cases	0	0
4. Not sure	2	7.69
10. Can you think of other areas where 3D printed models might be useful in your work?		
Yes	22	84.62
No	4	15.38
11. Are 3D printed models useful in the education and training of primary surgeons?		
Yes	22	84.62
No	0	0
Not sure	4	15.38

Abbreviation: 3D, Three‐dimensional.

**FIGURE 5:**
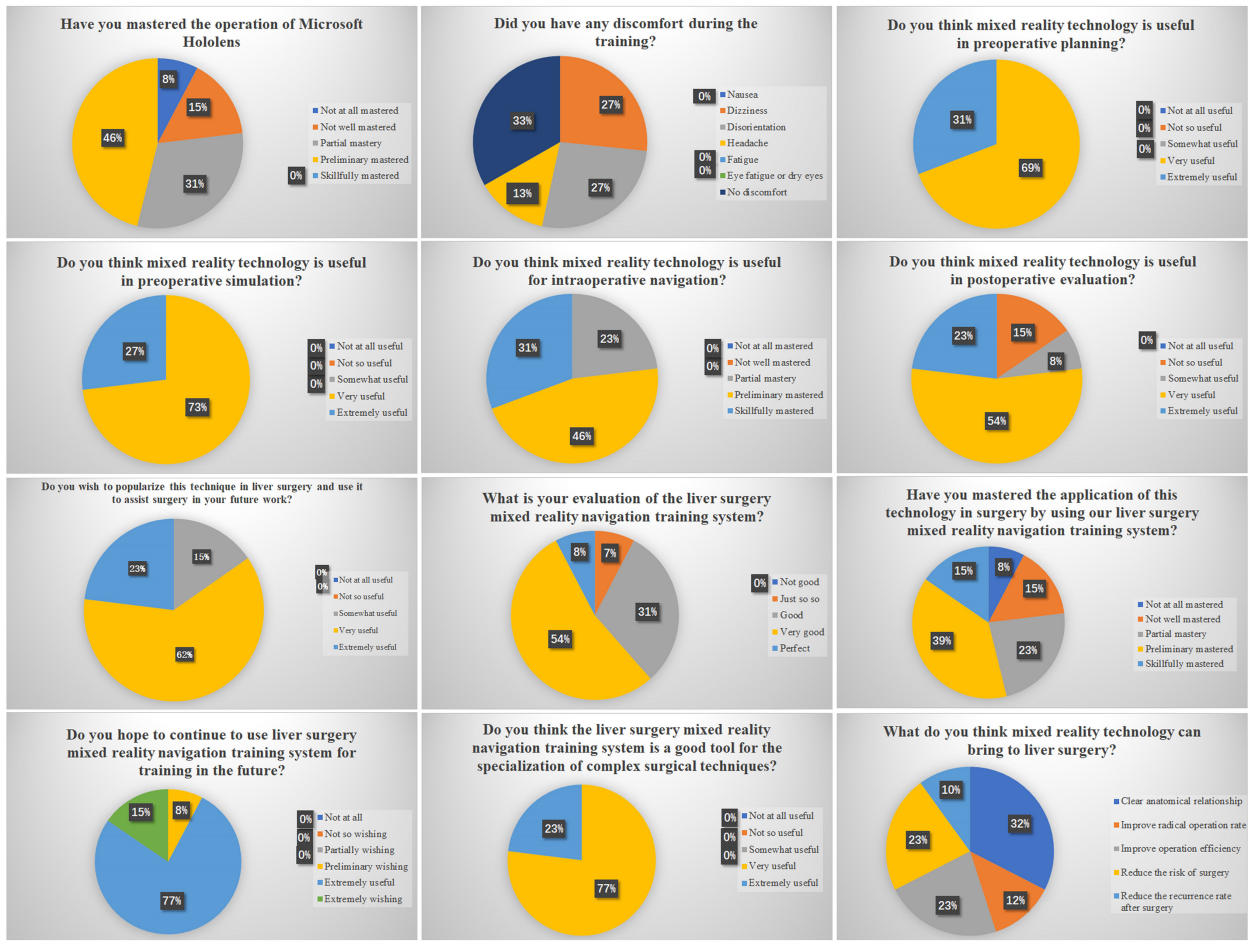
Evaluation of problems related to hybrid reality navigation training system in liver surgery

In the questionnaire of the liver surgery mixed reality navigation training system, 77% of the participants mastered the operation of HoloLens glasses through the training part. The Remaining 30.77% of the participants developed dizziness and disorientation, and 15.38% of the participants had a headache. All participants thought that mixed reality technology was useful in preoperative planning, simulation and intraoperative navigation. The 84.62% of participants thought it was useful in postoperative evaluation. The 84.62% participants hoped to promote mixed reality technology in future liver surgery. The 92.31% of the participants had a good evaluation of the hybrid reality navigation training system for liver surgery. The 76.92% of the participants mastered the application of this technology in surgery through our liver surgery hybrid reality navigation training system. All participants believed that the liver surgery hybrid reality navigation training system was a good tool for the specialization of complex surgical techniques. We hope to continue using our system for further training. It can clarify the anatomical relationship (32%), improve the radical operation rate (12%), improve the operation efficiency (23%), reduce the operation risk (23%), and reduce the postoperative recurrence rate (10%) (Figure 5).

Participants also put forward areas for improvement. They want the system to be most stable and easier. The glasses were too heavy. The change of position during the operation, would change image accordingly, suggesting to fix the position of the image. To design a training system to integrate laparoscopy and HoloLens glasses.

## 4 DISCUSSION

In 1997, Fasel et al.^5^ selected 148 cross‐sectional data with 1 mm slice thickness from the VHP data set to reconstruct the liver and part of the trunk of intrahepatic ducts. In 2005, Fang et al.^6^ selected 875 cross‐sectional data with thickness of 0.2 mm from the dataset of “Digital Virtual Chinese Female I". In 2009, Lou et al.^7^ selected 233 coronal data with thickness of 0.6 mm from the coronal data set. In 2009, Chen et al.^8^ selected 178 cross‐sectional data with a thickness of 1 mm from the Chinese digital human dataset. In 2009 Shin et al.^9^ selected 277 cross‐sectional data with a thickness of 0.2 mm from the visible Korean human dataset to reconstruct liver and intrahepatic duct. We found that with the passage of time, the accuracy and precision of reconstructions are getting higher and higher. First, the dataset obtained is better, which is manifested in thinner layer thickness, higher resolution and more accurate inter‐layer registration. Secondly, with the progress of segmentation and reconstruction software, the functions of software are becoming stronger, the efficiency is constantly improved, and new functions are constantly appearing. Of course, this is accompanied by the rapid development of computer hardware performance.^10^ We compare results of the previous reconstruction lists as follows. Tabular comparison was made between data parameters obtained in this study and previous studies on liver dissection (Table 3).

**TABLE 3:** Comparison of data parameters of the sectional anatomy of Liver studies between present study and Fasel et al., Fang et al., Lou et al., Chen et al. and Shin et al.^9^, ^10^, ^11^, ^12^, ^13^

Year	Author	Dataset name	Perfusion	Slice direction	Section	Thickness	Resolution	Number of slices	Segmentation method	Type of vessels	Reconstruction method
1997	Fasel et al.	VHP	No	Head‐foot	Transverse	1 mm	2048 × 1216	148	Manual	3	Surface rendering
2005	Fang et al.	VCH‐F1	Artery red	Head‐foot	Transverse	0.2 mm	3024 × 2016	875	Manual+Auto	4	Volume & surface rendering
2009	Lou et al.	CDH‐2	No	Front‐back	Coronal	0.2 mm	3504 × 2336	233	Manual	2	Volume & surface rendering
2009	Chen et al.	CVH‐1	Artery red	Head‐foot	Transverse	1 mm	3072 × 2048	178	Manual	4	Volume & surface rendering
2009	Shin et al.	VKH	No	Head‐foot	Transverse	0.2 mm	3040 × 2008	277	Manual	4	Surface rendering
2021	Shahbaz et al.	CDH‐F2	Artery red &Vein blue	Foot‐head	Transverse	0.1 mm	13,000 × 8000	1540	Manual	4	Surface rendering

Abbreviations: CDH‐2, Chinese Digital Human Number 2; CHD‐F2, Chinese Digital Human‐Female No. 2; CVH‐1, Chinese Visible Human Number 1; VCH‐F1, Virtual Chinese Human Female number 1; VHP, Visible Human Project; VKH, Visible Korean Human.

In order to ensure the effect, gelatin plus red dye or blue dye was used to make arterial or venous fillers for perfusion, so that the lumen of hepatic vein was blue and the proper hepatic artery was red. The hepatic duct was yellow because of the presence of bile. The hepatic portal vein had no special color. In this way, four kinds of intrahepatic ducts have identification basis. Although, perfusion cannot ensure that all the small branches could be reached. The continuous playback of pictures, real‐time tracking was used to ensure the identification of various ducts. The CNC milling machine used has the advantages of stable operation and high accuracy, which ensures the uniform layer thickness of the dataset 0.1 mm. Milling from the foot to the head, the cross section obtained was the lower surface of the specimen. It is consistent with the direction of clinical imaging examination.

In the process of image segmentation, we tried to use the threshold segmentation method, but the accuracy was not enough.^20^ The small ducts were difficult to identify automatically. The error rate was high. The four systems were easy to be confused. In order to get better effect and accuracy, we used manually segmented 1540 cross‐sectional data respectively. The workload was heavy. Because the distance between the adjacent layers was only 100 μm, and the corresponding structure changes little. We copied the paths of the previous layer to the next layer, and then made minor adjustments. It helped us to identify small ducts and greatly reduced the workload. The Digihuman 3D Reconstruction System is a special software for digital anatomy independently developed by Shandong Digital Man Company based on openCV. It optimizes the sectional anatomical segmentation and ensures the speed and stability of the reconstruction process.

The 3D model improves the teaching of anatomy and surgical residents' understanding of surgical anatomy. A better understanding of liver anatomy may contribute to laparoscopic or open hepatectomy.^21^, ^22^ Virtual reality produces an interactive 3D environment, which makes the real‐life experience realistic and immersive. Augmented reality provides enhanced Virtual Reality rendering, providing surgeons with basic information to optimize navigation during complex operations and reduce intraoperative and postoperative complications.^23^, ^24^ Mixed reality (MR) technology, as a new technology formed on the basis of 3D applications, overlays anatomical structures directly on the target organs, and it has high potential to improve the movement and perception of surgeons in open visceral surgery.^14^, ^25^ In abdominal surgery, the liver is deformed and results in change of form, position and size by respiration, pneumoperitoneum, body position, tissue dissection, traction and iatrogenic manipulation which requires the surgeon to make a judgment based on the actual situation during surgery.^26^, ^27^, ^28^, ^29^, ^30^ The purpose of our system training is to make surgeons more proficient in using mixed reality technology to assist them in making judgments and interpret and act upon any apparent error during surgery, a judgment that requires a combination of factors including prediction of the tissue deformation and use of advanced visualization algorithms to assist the surgeons to identify the overlay error.^31^

A study emphasized the advantages of using personalized 3D liver models to guide hepatectomy.^22^ We believe that the virtual effect of MR surgery is closely related to image scanning parameters. In order to establish a high‐precision model, we need high‐precision imaging data, otherwise the processed data is of little significance to clinical guidance.^31^ When compared with the results of 3D reconstruction of clinical imaging data, the complex details were absent. Our results provide a comprehensively detailed model of intrahepatic duct distribution. We can display the reconstructed model by 3D printing, Virtual Reality, Augmented Reality and MR.

MR exchanges information quickly through the connection between reality and virtual world, and enhances the reality of experience.^32^, ^33^ The three basic attributes of MR technology determine its unique advantages in clinical teaching: immersion, interaction, and imagination. In the field of abdominal surgery, mixed reality technology has been gradually applied and achieved good clinical results. A study applied mixed reality technology in laparoscopic nephrectomy, and achieved good results in operation plan, intraoperative navigation, remote consultation, teaching, and doctor‐patient communication etc.^34^ Our study shows the treatment process of liver tumor intuitively as a 3D spatial structure through MR technology. Our training system combines 3D printing technology and mixed reality technology to train surgeons in the operation of mixed reality technology. This greatly reduced the difficulty of identifying the complex spatial structure of the tumor. It helps to enhance the students' understanding of the operation plan, and significantly shortens the learning cycle.^35^ When the mixed reality technology is applied to the clinical teaching of liver surgery, students show active interest and higher initiative. The mixed reality technique was applied to the operation of liver tumor, and its application value in the operation of liver tumor was explained from the aspects of preoperative planning, teaching, and training, and the care of important anatomical structures intraoperatively (Figure S5).^36^, ^37^

We used HoloLens glasses combined with 3D model to build a training system. The preoperative reconstruction image is directly projected on the model, so that the operator can accurately predict the course of the hepatic duct, the location of the tumor and its relationships, and take this as the basis for excision. The main advantages of our study are; it can accurately locate the location of hepatic vessels and tumors before and during operation, preoperative simulation, determine the surgical incision and simulate the scope of resection, so as to achieve ideal surgical results. We are working to reconstruct the organs and blood vessels around the liver to form a virtual abdominal anatomy system with rich details, and combine 3D printing technology with mixed reality technology to improve the mixed reality navigation training system of liver surgery, to provide most guidance for surgery.^38^ This system is a training system based on the normal anatomy of the liver, and the training is intended to enable surgeons to achieve high levels of precision become proficient in the operation of mixed reality technology and avoid complications. Mixed Reality training is a worthy alternative to provide 3D information to clinicians and its application in surgery. This conclusion was obtained based on a questionnaire and evaluation. Surgeons with extensive experience in surgical operations perceived in the questionnaire that this technology is useful in liver surgery, would help in precise preoperative planning, accurate intraoperative identification, and reduction of hepatic injury.^39^, ^40^ MR technology can help physicians with precise positioning during surgery. However, the surgeon's proficiency in operating MR glasses has limited the diffusion of this technology. In the future, we will reconstruct data and 3D print isometric models based on CT data of liver tumors to be applied to our training system. In actual surgery, it is difficult to render the finer vessels with CT data. Our model reconstructed based on tomographic anatomical data can allow doctors to see more details, different from the actual CT, but we believe the trainers will have different gains. In future studies we plan to improve registration accuracy and non‐rigid registration algorithms will be required to address intraoperative anatomical deformation.^32^, ^41^, ^42^, ^43^, ^44^

Our study has several limitations. The 3D reconstruction and modeling of cross‐sectional anatomical dataset was from one cadaver. Dataset was obtained manually which is time consuming and 3D printed model is costly. There are potential inaccuracies at each stage of model fabrication as well.^12^ The training of “MRNSALTS” System was conducted by a group of 26 clinicians which need to be enlarged.

## 5 CONCLUSION

This study shows that a higher quality cross‐sectional anatomical dataset can reconstruct detailed 3D models. A hybrid reality navigation training system for liver surgery is created by the combination of 3D printing and HoloLens glasses virtual reality technology. Mixed Reality training is a worthy alternative to provide 3D information to clinicians and its possible application in surgery. This conclusion was obtained based on a questionnaire and evaluation. Surgeons with extensive experience in surgical operations perceived in the questionnaire that this technology might be useful in liver surgery, would help in precise preoperative planning, accurate intraoperative identification, and reduction of hepatic injury.

## AUTHOR CONTRIBUTIONS

**Muhammad Shahbaz:** Conceptualization (lead); data curation (lead); formal analysis (equal); investigation (equal); methodology (lead); resources (equal); software (equal); validation (equal); visualization (equal); writing – original draft (lead); writing – review and editing (lead). **Huachun Miao:** Conceptualization (lead); data curation (equal); formal analysis (equal); investigation (equal); methodology (lead); software (lead); writing – original draft (equal); writing – review and editing (equal). **Zeeshan Farhaj:** Formal analysis (equal); investigation (equal); methodology (equal); software (equal); writing – original draft (equal); writing – review and editing (equal). **Xin Gong:** Conceptualization (equal); data curation (equal); formal analysis (equal); investigation (equal); methodology (equal); software (equal); writing – original draft (equal). **Sun Weikai:** Data curation (equal); formal analysis (equal); investigation (equal); methodology (equal); resources (equal); software (equal); writing – original draft (equal). **Wenqing Dong:** Data curation (equal); formal analysis (equal); investigation (equal); methodology (equal); resources (equal); software (equal); writing – original draft (equal). **Niu Jun:** Conceptualization (equal); methodology (equal); project administration (equal); resources (equal); software (equal); supervision (equal); validation (equal); writing – original draft (equal). **Liu Shuwei:** Conceptualization (equal); data curation (equal); funding acquisition (equal); methodology (equal); project administration (equal); resources (equal); software (equal); supervision (equal); validation (equal); writing – original draft (equal); writing – review and editing (equal). **Dexin Yu:** Conceptualization (equal); data curation (equal); formal analysis (equal); funding acquisition (equal); methodology (equal); project administration (equal); resources (equal); software (equal); supervision (equal); validation (equal); writing – original draft (equal); writing – review and editing (equal).

## FUNDING INFORMATION

This work was supported by Major scientific and technological innovation projects Shandong Province (No. 2015ZDXX0201A02, No. 2019JZZY020106), National Natural Science Foundation China (No. 81771888) and Shandong Provincial Natural Science Foundation China (ZR2017MH006).

## CONFLICT OF INTEREST

The authors declare no conflict of interest.

## ETHICS STATEMENT

This study was approved by the Ethics Committee of Basic Medical Sciences, Shandong University (IRB No. ECSBMSSDU2018‐1‐050 and Ethics Committee of Scientific Research of Shandong University Qilu Hospital IRB No. KYLL‐2022(ZM)‐749).

## Supporting information


Figure S1.
Figure S2.
Figure S3
Figure S4
Figure S5

Video S1

## Data Availability

The data that support the findings of this study are available from the corresponding author upon reasonable request.
