# Supplementary material for: Mixed reality navigation training system for liver surgery based on a high‐definition human cross‐sectional anatomy data set
Source: Cancer Med. 2023 Jan 6;12(7):7992–8004. doi: 10.1002/cam4.5583 (PMC10134360; doi:10.1002/cam4.5583)
Supplement: Supplementary file 1 — Figure S1. Figure S2. Figure S3 Figure S4 Figure S5 [file CAM4-12-7992-s002.docx]

**Supplementary Fig 1.** Reconstruction of Liver (**A** front view, **B** rear view), Reconstruction of liver and hepatic vessels (**C** front view, **D** rear view), **E** Reconstruction of proper hepatic artery, **F** Reconstruction of hepatic duct, **G** Reconstruction of hepatic portal vein, **H** Reconstruction of hepatic vein


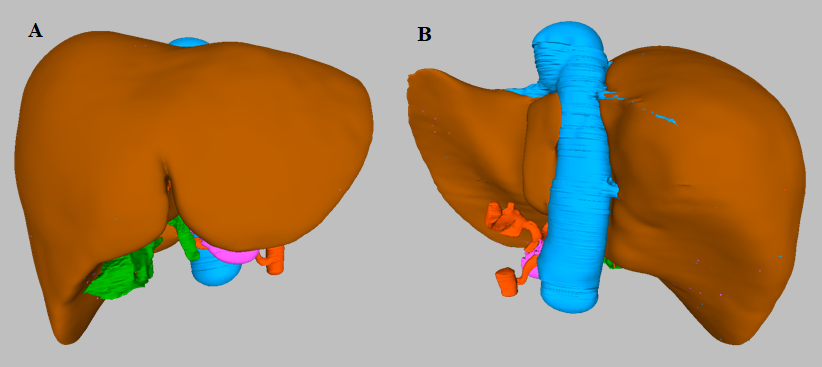


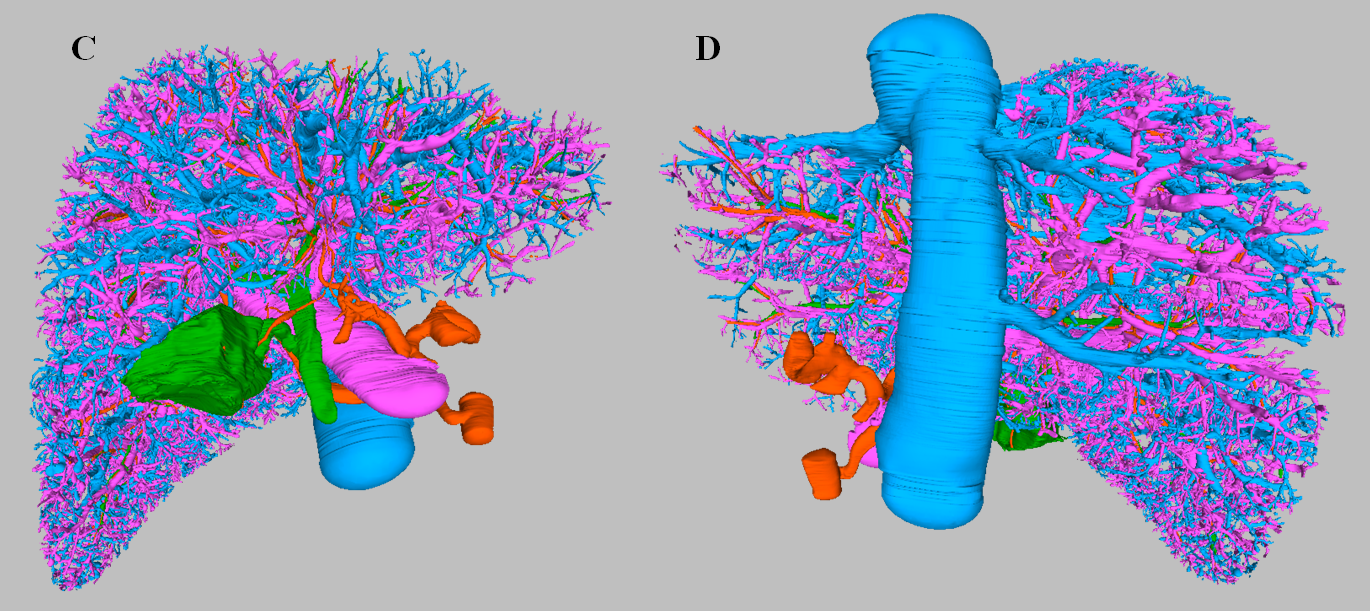


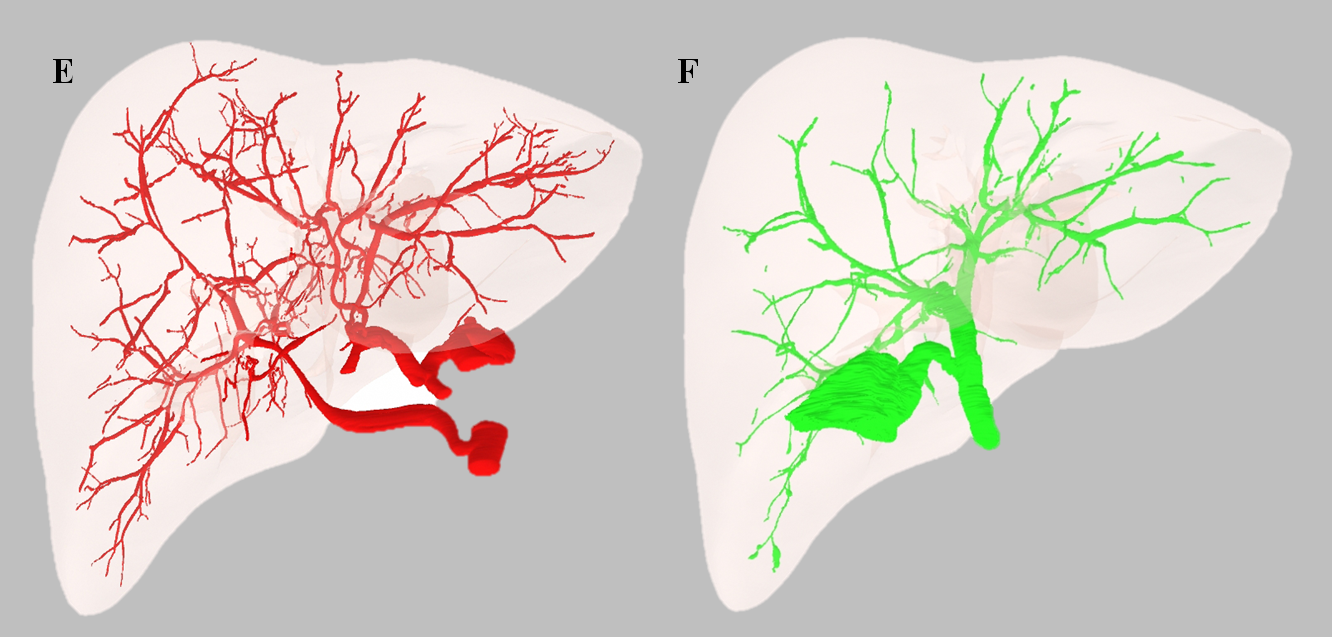


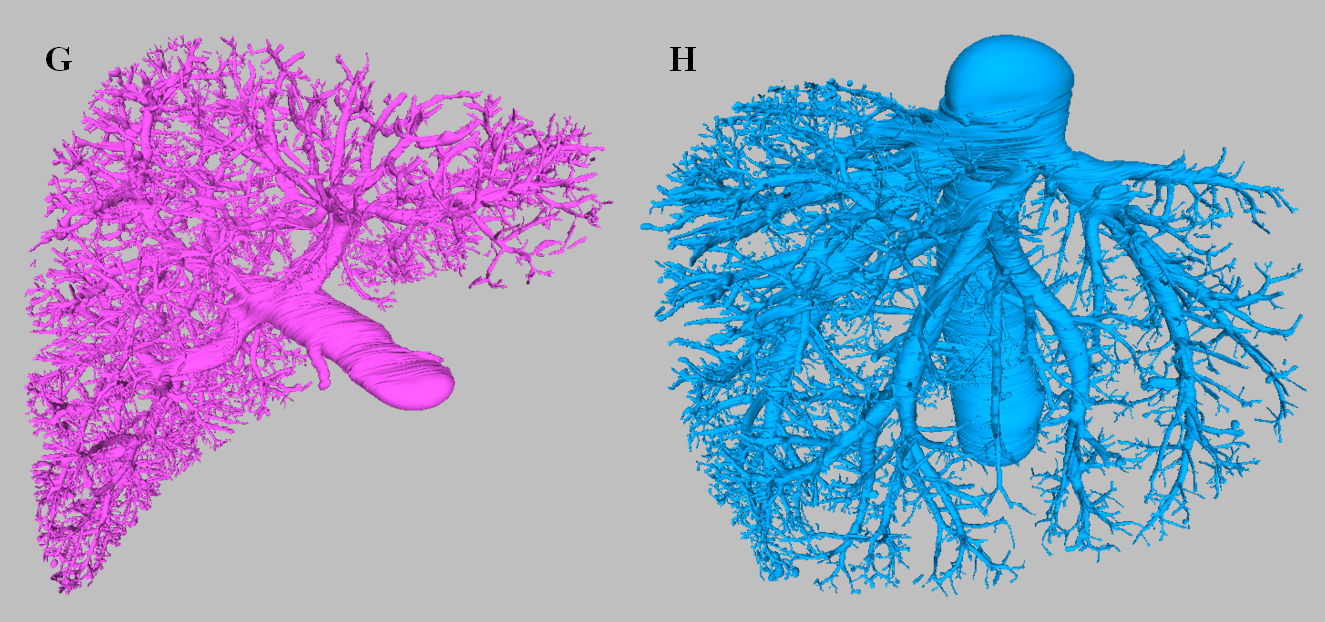


**Supplementary Fig 2.** The reconstructed model of hepatic duct: **A** hepatic duct in liver (1.common bile duct, 2.common hepatic duct, 3.gallbladder, 4.right hepatic duct, 5.left hepatic duct, 6.right posterior branch, 7.right anterior branch, ***point*** 8.right posteroinferior branch, ***point*** 9.right posterosuperior branch, ***point*** 10.right anteroinferior branch, ***point*** 11.right anterosuperior branch, ***point*** 12.left laterosuperior branch, ***point*** 13.left lateroinferior branch, ***point*** 14.left medial branch, ***point*** 15.caudate branch). **B** biliary tree


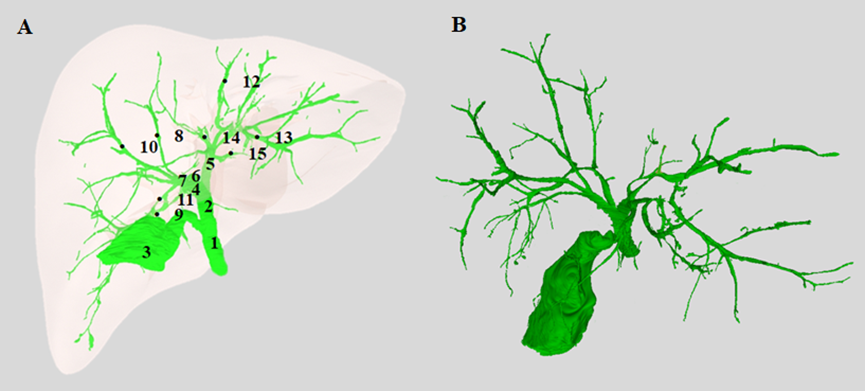


**Supplementary Fig 3.** The reconstructed model of hepatic portal vein: **A** front view, **B** rear view(1.left branch,2. right branch,3. right posterior branch,4. right anterior branch,5. right posteroinferior branch,6. right posterosuperior branch,7. right anteroinferior branch,8. right anterosuperior branch,9. left laterosuperior branch,10. left lateroinferior branch,11. left medial branches, ***point*** 12. caudate branches)

**
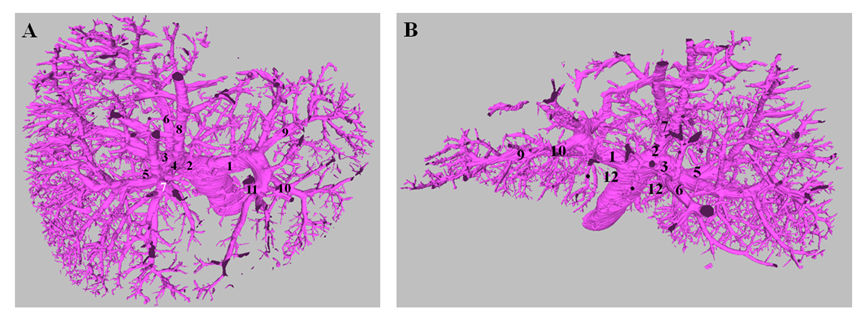
**

**Supplementary Fig 4.** The reconstructed model of hepatic vein: **A** integral view, **B** accessory hepatic vein (1.left hepatic vein, 2.intermediate hepatic vein, 3.right hepatic vein, 4.left posterior supramarginal vein, 5.left interlobar vein, 6.right front branch from segment VIII ventral, 7.right front branch from segment VIII dorsal, 8.right posterior supramarginal vein, ***point*** 9. Caudate hepatic vein, ***point*** 10. Right posterior hepatic vein


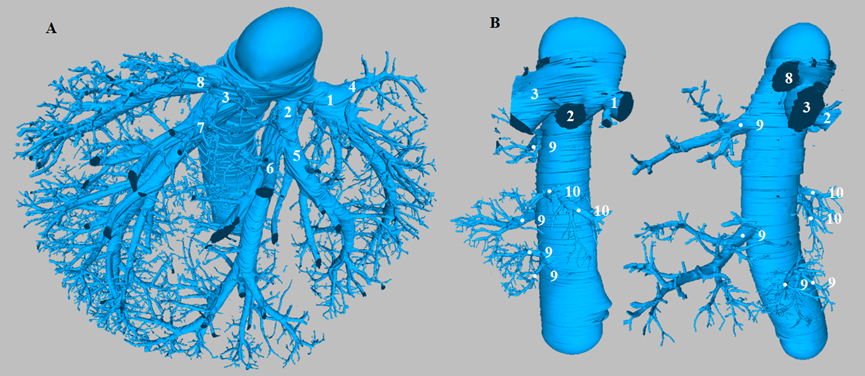


**Supplementary Fig 5. 3D reconstruction model of a patient with Hepatocellular carcinoma**

**
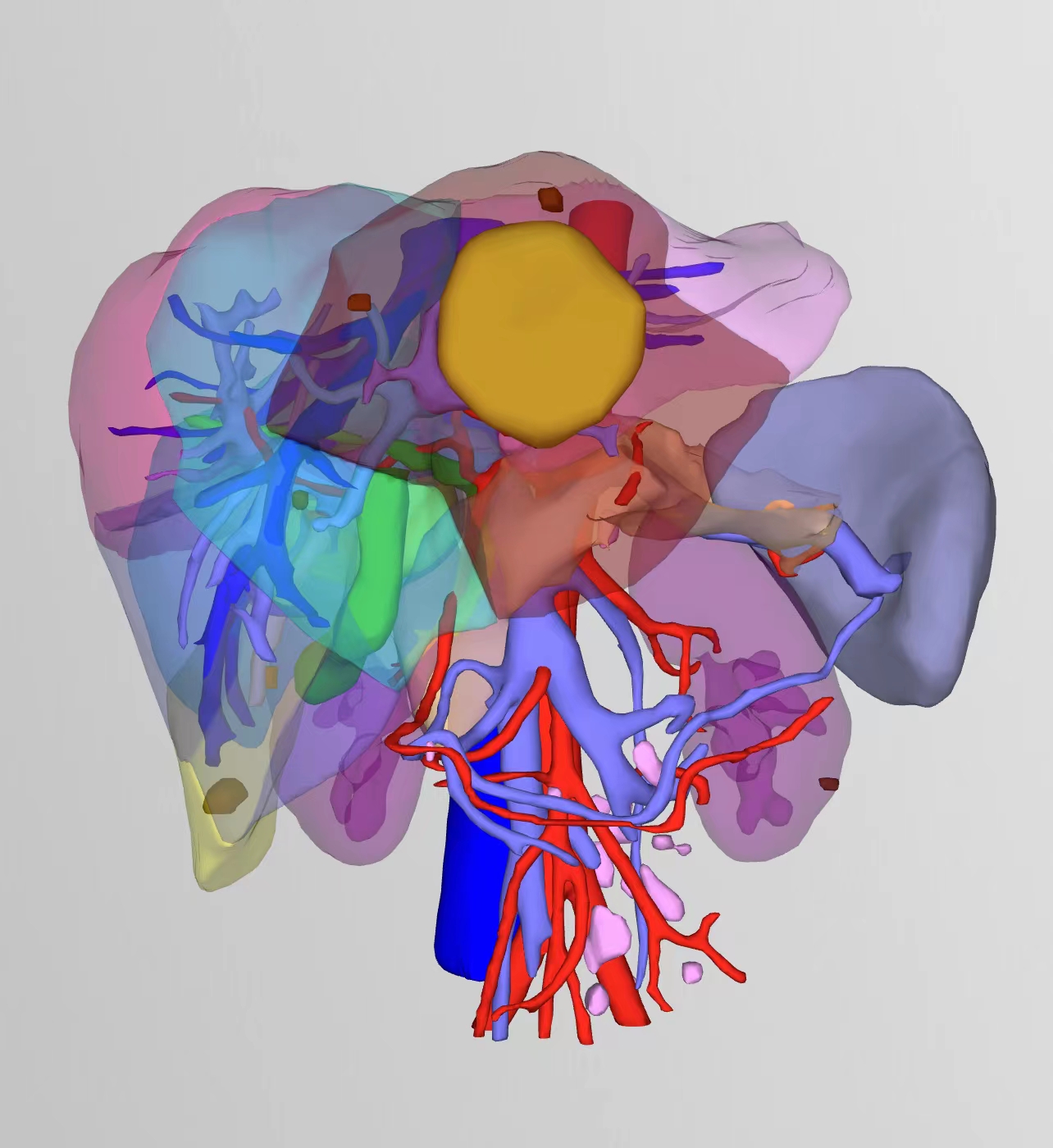
**
